# Bioactive Potential of Carrot-Based Products Enriched with *Lactobacillus plantarum*

**DOI:** 10.3390/molecules29040917

**Published:** 2024-02-19

**Authors:** Monica Boev, Cristina Stănescu, Mihaela Turturică, Mihaela Cotârleţ, Denisa Batîr-Marin, Nicoleta Maftei, Carmen Chiţescu, Leontina Grigore-Gurgu, Vasilica Barbu, Elena Enachi, Elena Lăcrămioara Lisă

**Affiliations:** 1 Department of Pharmaceutical Sciences, Faculty of Medicine and Pharmacy, Dunărea de Jos University, 800008 Galati, Romania; monica.boev@ugal.ro (M.B.); denisa.batir@ugal.ro (D.B.-M.); nicoleta.aron@ugal.ro (N.M.); carmen.chitescu@ugal.ro (C.C.); elena.lisa@ugal.ro (E.L.L.); 2 Department of Morphological and Functional Sciences, Faculty of Medicine and Pharmacy, Dunărea de Jos University, 800008 Galati, Romania; dr.cristinastanescu@gmail.com; 3 Department of Food Science, Food Engineering, Biotechnologies and Aquaculture, Faculty of Food Science and Engineering, Dunărea de Jos University, 800008 Galati, Romania; mihaela.turturica@ugal.ro (M.T.); mihaela.cotarlet@ugal.ro (M.C.); leontina.gurgu@ugal.ro (L.G.-G.); vasilica.barbu@ugal.ro (V.B.)

**Keywords:** carrot, lactic acid bacteria, carotenoids, cubes, chips, powders

## Abstract

The primary goal of this study was to generate different kinds of functional products based on carrots that were supplemented with lactic acid bacteria. The fact that carrots (*Daucus carota* sp.) rank among the most popular vegetables in our country led to the convergence of the research aim. Their abundance of bioactive compounds, primarily polyphenols, flavonoids, and carotenoids, offers numerous health benefits. Among the obtained products, the freeze-dried carrot powder (*FDCP*) variation presented the highest concentrations of total carotenoids (*TCs*) and *β*-carotene (*BC*) of 26.977 ± 0.13 mg/g DW and 22.075 ± 0.14 mg/g DW, respectively. The amount of total carotenoids and *β*-carotene significantly increased with the addition of the selected lactic acid bacteria (LAB) for most of the samples. In addition, a slight increase in the antioxidant activity compared with the control sample for the *FDCP* variant, with the highest value of 91.74%, was observed in these functional food products. The content of polyphenolic compounds varied from 0.044 to 0.091 mg/g DW, while the content of total flavonoids varied from 0.03 to 0.66 mg/g DW. The processing method had an impact on the population of *L. plantarum* that survived, as indicated by the viability of bacterial cells in all the analyzed products. The chromatographic analysis through UHPLC-MS/MS further confirmed the abundance of the bioactive compounds and their corresponding derivatives by revealing 19 different compounds. The digestibility study indicated that carotenoid compounds from carrots followed a rather controlled release. The carrot-based products enriched with *Lactobacillus plantarum* can be considered newly functional developed products based on their high content of biologically active compounds with beneficial effects upon the human body. Furthermore, these types of products could represent innovative products for every related industry such as the food, pharmaceutical, and cosmeceutical industries, thus converging a new strategy to improve the health of consumers or patients.

## 1. Introduction

*Daucus carota* sp. is part of the *Umbelliferae*, *Apiaceae* family, can be consumed raw, cooked, or in juices, and is one of the most appreciated vegetables. Carrots present a high amount of proanthocyanidins, saponins, fiber content, and total polyphenol, total flavonoid, and total carotenoid contents [1,2,3]. It also has biological activities, antioxidant, anticancer, and cardiovascular disease-preventive effects, and reduces degenerative disease development [3,4]. Several carrot varieties display different colors, shapes, and sizes, the most common color being orange, but there are also white, yellow, red, purple, and black carrots [5]. The world production of carrots has increased over recent years, reaching 44.76 M tons in 2019. The major producers are China (21.38 M tons), Uzbekistan (2.77 M tons), USA (2.26 M tons), Ukraine (869,450 tons), UK (83,0259 tons), Germany (791,110 tons), Indonesia (698,880 tons), and Poland (678,300 tons) [6]. Asia and Europe have the highest production share in carrots, with 29.03 M tons and 8.53 M tons, respectively [6].

An important agro-food waste in Romania is carrot pulp. This type of vegetable waste is well known to have high amounts of polyphenolic compounds with a high antioxidant potential and dietary fibers [7,8,9]. For example, carotenoid compounds are directly responsible for the color, aroma, and bitterness of carrots [10]. Moreover, according to Arscott et al. [11], carrot phenolic acids have been proven to reduce cardiovascular diseases.

According to Otoni et al. [12], approximately 70–75% of harvested carrots are marketed after minimal processing as Baby Carrots™, carrot chips, and sticks, or as chopped, shredded, and sliced carrots. Nonetheless, the proper extraction of the biologically active compounds leads to new directions that take into consideration the alternative application of this vegetable in functional foods [7], medicines [13], pharmaceuticals, and cosmetics [14]. An important constituent found in carrot peel is pectin (E440), a soluble dietary fiber, used as an additive in functional foods (frozen foods, jams, etc.) that exhibits the capability to form a dense gel, so it is perfect to replace hydrocolloids [8]. Carrot fibers present high antioxidant but also prebiotic features [15,16]. Other valuable and also important constituents present in the carrot peel are carotenoids (α- and *β*-carotene) [4]. Commercial carrot essential oils are well perceived by the flavor industry, not only because of their strong aroma but also because of their antibacterial and fungicidal activities, which make them a good candidate for food preservation [17].

The first vegetable recognized as safe for use in the biopharmaceutical industry was the carrot [1]. The consumption or the enrichment of food products with carrot by-products has a good impact on human health with several benefits such as decreasing blood pressure and the prevention of heart diseases, atherosclerosis, infections, bronchial asthma, and also muscular degeneration. Polyphenolic compounds found in carrot peel have antioxidant, antimutagenic, and antitumor actions, and their dietary fibers prevent heart diseases, constipation, and cancer and control blood sugar levels as therapeutical molecules [1,18,19]. Probiotics have been predominantly used in dairy products since dairy products provide a favorable culture medium for probiotic viability [20]. Thus, ≈78% of annual probiotic sales worldwide correspond to the dairy market [21]. The demand for non-dairy products with probiotics has emerged due to technological improvements, as a new opportunity for the development and delivery of functional foods, and thus, there is an increase in availability in the market, making this food segment have promising interest from different consumers. Specifically, Lillo-Pérez et al. [22] estimated that the market for probiotic products of non-dairy origin grew by 15% (every year) between 2013 and 2018 since the producers manufactured new tastier and healthier products that are much more attractive to all types of consumers, accepting this type of product as being healthy and refreshing. In their study, Shigematsu et al. [23] developed an edible coating based on sodium alginate that has probiotic potential, and they determined the viability of *L. acidophilus* in slices of minimally processed carrots that were stored for 19 days at 8 °C. Also, de Oliveira et al. [24] reported the use of *L. rhamnosus*, *L. plantarum*, and *L. acidophilus* in mango and carrot mixed juices, and the authors showed good viability in juices with a high amount of carrot, probably due to the higher fiber concentration of carrots.

According to the latest studies conducted by Barbu et al. [25], lactic acid bacteria (LAB) play an essential role in improving the microbiological quality and in expanding the shelf life of many functional food products. Also, the need for consumers to benefit from innovative functional products highlights the potential of these types of products. As such, this study presents, as the most important objective, the analysis of three types of carrot products enriched with LAB to highlight their functionality and their nutritional value.

## 2. Results and Discussion

### 2.1. Survival of L. plantarum in Carrot-Based Products

Several factors could influence the stability of probiotics in food products during production and storage, such as strains of probiotic bacteria, moisture content, storage temperature, and packaging materials [16]. The viability of the bacterial cells from all analyzed products indicated that the population of surviving *L. plantarum* was affected by the processing method. The initial LAB count was evaluated. For fresh carrot cubes (*FCCs*), a viability of 8.04 log^10^ CFU/g was calculated, while for dried carrot chips (*DCCs*) and freeze-dried carrot powder (*FDCP*), survival rates of 8.53 and 8.59 log^10^ CFU/g were obtained. These values indicated good adaptation and attachment of LAB on the carrot-based products’ surfaces. Akman et al. [26] reported an initial inoculation level of 7.69 log^10^ CFU/g for *L. paracasei* on dried apple slices.

**Figure 1:**
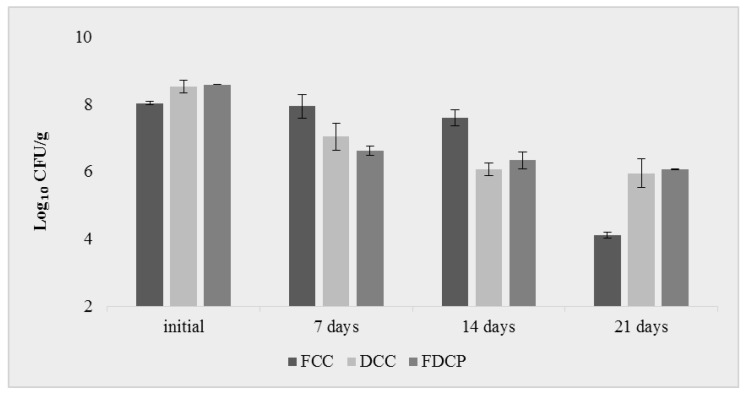
Viability of *L. plantarum* MIUG BL4 during 21 days of storage: *FCCs*—fresh carrot cubes; *DCCs*—dried carrot chips; *FDCP*—freeze-dried carrot powder (data are the means ± SD).

The microbiological analysis showed that during storage for all analyzed samples, the number of probiotic lactic acid bacteria was reduced by 0.44 log^10^ (for *FCCs*), 2.46 log^10^ (for *DCCs*), and 2.26 log^10^ (for *FDCP*) after 14 days (Figure 1) but still conserved an amount of cells of 6.0–7.0 log^10^ CFU/g). It can be concluded that in all carrot-based products, the survival of *L. plantarum* MIUG BL4 was below 6.0–7.0 log^10^ CFU/g after 14 days of storage, which could be recognized as the shelf life of these functional products. The results were in accordance with Barbu et al. [25], who reported values of *L. plantarum* MIUG BL3 survival between 6.71 log^10^ CFU/g in fresh cubes of beetroot and 7.84 log^10^ CFU/g in dried beetroot chips. 

Liu et al. [16] recommended that vegetal-based products should be supplemented with a higher number of probiotic cells. Even though the minimum therapeutic dosage is unclear, the concentration of 6.0–7.0 log^10^ CFU/g or mL of products is typically suggested [25].

The survival rates in all samples could be explained by the fact that the vegetal matrix protected and microencapsulated the bacterial cells, thus maintaining their viability. Freeze-drying better preserved the morphology of probiotic microcapsules [25].

Although freeze-drying is an expensive and slow dehydration technique, it has been widely used to obtain high-quality and high-value dehydrated fruits and vegetables [25].

### 2.2. Scanning Electron Microscopy

**Figure 2:**
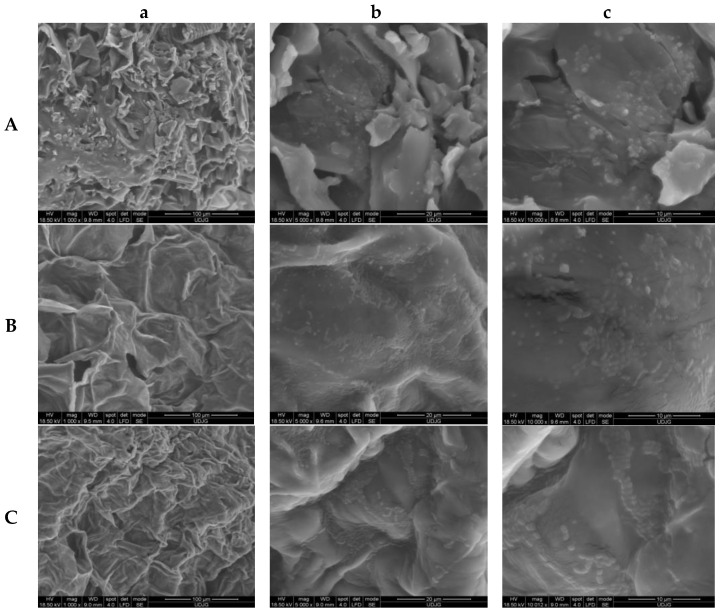
SEM images of samples ultrastructure: (**A**)—freeze-dried carrot powder (*FDCP*); (**B**)—fresh cubes with LAB (*FCCs*); (**C**)—dried chips with LAB (*DCCs*); a—1000×; b—5000×; c—10,000×.

The scanning electron microscopy (SEM) analysis of the experimental variants showed parenchymatic tissue with flattened cells, deformed to varying degrees depending on the obtainment technology. Although the drying temperature was low (40 °C) in order to protect the biologically active compounds in the plant cells, in the *DCC* variant (Figure 2Ca), they did not maintain their turgidity. In the *FDCP* variant (Figure 2Aa), it can be seen that the cell walls were broken and fragmented. The probiotic LAB strain *L. plantarum* MIUG BL4 used to obtain the experimental variants adhered very well to the cell substrate, thus forming extensive biofilms in which the bacilli manifested an aggregative adhesion phenotype. The adhesion pattern was similar, with cells grouped in chains and palisades, visible in all the samples, especially at a magnification of 10,000× (Figure 2Cc). This aggregative characteristic of the starter strain is due to its ability to develop exopolysaccharides (*EPSs*) that mediate the interaction between the cells of the biofilm and also between them and the cell substrate [27]. The more drastic the technological conditions (*DCC* variant Figure 2Cc), the thicker the *EPS* capsule of the lactic acid bacteria in the biofilm as an adaptation to them. The fresh sample (*FCCs*) offered the most favorable environment for the multiplication of the lactic acid bacteria, obviously due to the accessibility of the nutrients and humidity, so the biofilms had the largest surface (Figure 2Bc), thus presenting a more uniform adsorption of the probiotic lactic acid bacteria. The results differed by a small percentage to those obtained by Barbu et al. [25]. The communication among the live lactic acid bacteria mediated by quorum sensing, a process that may regulate gene expression, also may contribute to the health benefits to the consumers [28,29].

### 2.3. Confocal Laser Scanning Microscopy

**Figure 3:**
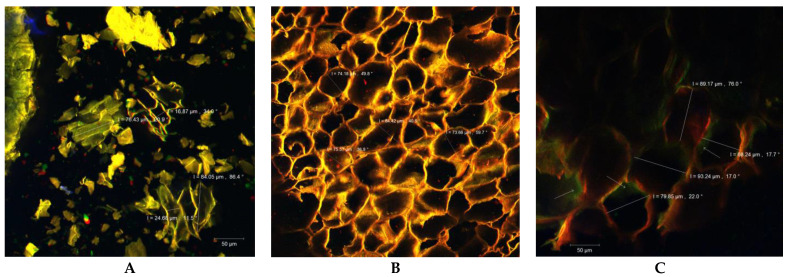
CLSM images of sample microstructures: (**A**)—freeze-dried carrot powder (*FDCP*); (**B**)—fresh cubes with LAB (*FCCs*); (**C**)—dried chips with LAB (*DCCs*).

The analysis of the experimental variants’ microstructures proved the maintenance in variable proportions of the vegetal tissue fragments from the metamorphosed carrot root. Isodiametric parenchymal cells with dimensions between 60 and 90 µm, with or without intact cell walls, could be visualized, obviously much better preserved in the *FCC* sample (Figure 3B). In the *FDCP* variant, cell debris was less frequent, while the cells were small and flattened due to the lyophilization process. Their dimensions were between 16.87 and 24.68/76.43 and 84.05 µm (Figure 3A). The presence of plant matrix residues, through the fiber content, adds functional value to the experimental variants. Inside the cells, the presence of carotenoids with a fluorescent emission in the range of 500–550 nm could be also observed (see arrows in Figure 3C). The viability of the lactic acid bacteria was best maintained in the *FCC* variant (Figure 3B) in which even the microcolonies of *L. plantarum* MIUG BL4, alive (in green) and dead (in red), could be visualized by using the fluorescent dyes included in the LIVE/DEAD BacLight kit (Thermo Fisher, Waltham, MA, USA). This result was also correlated with the microbiological analysis of the samples and could be explained by the multiplication of LAB in an environment rich in carbohydrates and biologically active compounds, with high humidity represented by fresh carrot cubes.

### 2.4. Carotenoid Quantification, Total Polyphenol Content, Total Flavonoid Content, and Antioxidant Activity

The total carotenoid (*TC*) and *β*-carotene (*BC*) contents in the fresh carrot cubes without LAB (control samples) were 4.466 ± 0.012 mg/g DW and 4.163 ± 0.031 mg/g DW, respectively (Table 1), *β*-carotene representing 93.22% of the total carotenoids. The *FDCP* variant without LAB registered significantly higher *TC* (21.609 ± 1.673 mg/g DW) and *BC* (20.705 ± 1.501 mg/g DW) contents compared to the *FCC* variant, while the *DCC* variant presented the lowest values. When compared to their corresponding controls, significant differences were observed, the *FDCP* sample displaying contents of 26.977 ± 0.130 mg/g DW for total carotenoids and 22.075 ± 0.14 mg/g DW for *β*-carotene. For the *FCC* variant, the *TC* and *BC* contents were 4.899 ± 0.017 mg/g DW and 4.620 ± 0.021 mg/g DW, whereas the *DCC* variant had a *TC* content of 0.534 ± 0.011 mg/g DW and a *BC* content of 0.492 ± 0.013 mg/g DW. The addition of the selected LAB caused a significant increase in the total carotenoid and *β*-carotene contents, especially in the *FDCP* variant, probably due to the increased extractability of these pigments from the matrix. The study conducted by Konopacka et al. [30] highlighted a total carotenoid content of 0.125 ± 0.006 mg/g DW in the case of dried carrot slices without any pretreatment.

According to Cui et al. [31], in their studies, total carotene losses in the hot-air-dried variants were encountered, and the causes of the loss of carotene content were heat, oxygen, and lipoxygenases. Konopacka et al. [30] stated that the carrot slices’ exposure to thermal processes (e.g., hot-air drying) led to a dehydration process of the tissue matrix, this process being the major cause of the carotenoid degradation, due to the *β*-carotene isomerization and other oxidation reactions that can highly decrease the biological activity of the final products [32]. In the freeze-drying process, because air and light were absent and the temperature used was very low, this could lead to an inhibition of oxidation and, therefore, the bioactive contents in the product could be considerably preserved [32,33].

In the case of carrots, the antioxidant activity is usually correlated with the *β*-carotene and the *TPC* contents. In this case, the highest value was registered for the *FDCP* sample while the lowest value was assessed for the *FCC* sample. The results of the antioxidant activity were well correlated with the *TPC* contents. Although it is known that processed fruits and vegetables might have lower antioxidant activity compared to raw or fresh due to vitamin C degradation during processing [30], drying at a mild temperature did not drastically change the antioxidant activity of the *DCC* variant. Our functional food products registered a slight increase in antioxidant activity compared to control samples, with the *FDCP* displaying the highest value, 91.74% (Table 1). The content of total polyphenols varied from 0.044 to 0.097 mg/g DW (Table 1). The content of total flavonoid content varied from 0.03 to 0.66 mg/g DW (Table 1).

The proanthocyanidin content of carrot samples increased as follows: hot-air drying > freeze-drying > fresh. The carrot sample that was hot-air-dried (0.33 mg *CE*/g DW) had the highest content of proanthocyanidins. Using freeze-drying slightly decreased the proanthocyanidin content to 0.209 mg *CE*/g DW. In the case of the *FCC* samples, when compared to the control, the results presented significant differences in the content of proanthocyanidins (0.089 and 0.094 mg *CE*/g DW, respectively). Our results are in accordance with the studies conducted by Nguyen et al. [3,14] on the *Phyllanthus amarus* and Xao tam phan (*Paramignya trimera*) roots, which highlighted that the proanthocyanidin content was the highest in the case of hot-air drying.

The highest content of saponins was obtained in the case of the *FDCP* sample (423.33 mg *EE*/g DW) and significant differences were found when compared to the control matrix’s results. The *FCC* sample displayed no significant differences when compared to its control variant (281.304 and 282.345 mg *EE*/g DW). So, the addition of the LAB had no impact on the saponin content when it came to the fresh variant, but instead for the other two variants, it can be seen that the factor that influenced the saponin content was the drying method. Saponins are secondary plant metabolites with therapeutic actions. In recent years, research has shown that saponins can be used as potential chemotherapeutic agents [34]. Therefore, our results regarding the *FCC*, *DCC*, and *FDCP* samples show that carrots are a promising source for developing functional foods.

### 2.5. The Chromatographic Analysis of the Biologically Active Compounds from the Carrot Extract

In order to characterize the experimental variants, a chromatographic analysis was performed in order to assess the presence of the biologically active compounds in carrots. The chromatographic profile of the carrot extract revealed the presence of several biologically active compounds, mainly phenolic acids, flavonoids, and carotenoids and their corresponding derivatives (Table 2). The compounds that recorded the highest concentrations were chlorogenic/neochlorogenic acid (148.75 ± 13.24 μg/g DW), rutin (quercetin 3-rutinoside) (102.45 ± 12.36 μg/g DW), naringin (92.77 ± 5.33 μg/g DW), and ferulic acid (85.24 ± 1.09 μg/g DW), whereas the concentrations for quercetin and catechin were 74.12 ± 13.99 μg/g DW and 63.45 ± 7.22 μg/g DW, respectively. *β*-carotene presented a concentration of 67.66 ± 8.02 μg/g DW while all the other compounds presented much lower concentrations. No levels could be identified for inulin, sinapic acid, coumaroylquinic acid derivative, hidroxyferulic acid, luteolin-O-glucoside derivative, procyanidine B1/B2, and procyanidine in the carrot extract because no reference substances were available. Nonetheless, their presence was assessed based on the theoretical and experimental different adducts and molecular masses. These results are comparable with those obtained by Lau et al. [32] on carrot peel extract and Nguyen et al. [35].

Maurer et al. [36] conducted a similar chromatographic analysis on an extract obtained from retail-purchased carrots. The primary carotenoids identified were *α*-carotene (45.00 ± 2.31 μg/g FW), *β*-carotene (53.58 ± 2.76 μg/g FW), and lutein (1.51 ± 0.09 μg/g FW). The values for lutein and *β*-carotene were consistent and ranged from 0.02 to 5.1 μg/g FW and 41.6 to 71.6 μg/g FW, respectively. The main differences could be from varietal and seasonal differences that can impact the biologically active compound content.

### 2.6. The Impact of Gastric and Intestinal Digestion on the Total Content of Carotenoids and β-Carotene from the Carrot Products

The in vitro digestibility analysis of the carrot products was used to evaluate carotenoid behavior in both gastric and intestinal juices. Prior to the absorption, according to the study conducted by Del Rio et al. [33], the bioactive compounds could be hydrolyzed in the gastrointestinal tract due to the environmental impact or due to some enzymes that are able to hydrolyze these compounds.

**Figure 4:**
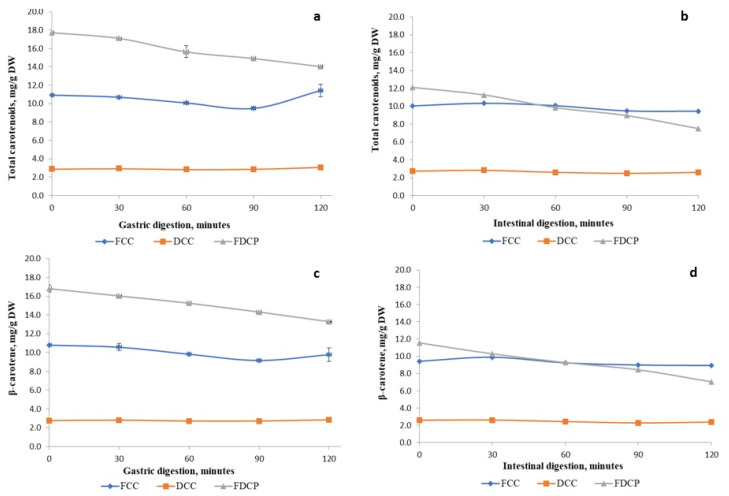
The digestibility of the carrot products’ total carotenoid (*TC*) and *β*-carotene (*BC*) content during gastric digestion (**a**,**c**) and intestinal digestion (**b**,**d**).

The action of the bioactive compounds in the gastrointestinal tract (Figure 4a–d) on human health represents a complex process that has a major effect on their release and absorption at the gastrointestinal level. In the simulated gastric juice, for the *FDCP* variant, a decrease in the carotenoid content was observed, with a percentage of 11.77% after 60 min of digestibility and around 20.79% after 120 min. Also, for the *FCC* variant, a decrease of 13.27% of the carotenoid content was observed after 90 min, then an increase of 4.5% after 120 min. In the simulated intestinal juice, there was an increase in the release of the carotenoids with values three times higher after 30 min of intestinal digestion, followed by a decrease of about 6% after 120 min for the *FCC* variant, while for the *FDCP* variant, there was a decrease of 38% after 120 min, thus suggesting a controlled release of these polyphenolic compounds. Before the in vitro digestibility process, the *FDCP* sample presented the highest content of carotenoids compared to the *FCC* and *DCC* samples, considering that the thermal processing, along with the microstructural changes related to the reduction in particle size, broke the cells and favored the release and bioavailability of carotenoids. Moreover, during both the gastric and intestinal steps, the total carotenoids were clearly degraded, an effect that was emphasized by the decreasing trend line highlighted in Figure 4a,b, the *FDCP* sample, and significant differences between the values were assessed. The carotenoids from the *FCC* variant, compared to *FDCP*, were continuously released from the carrot matrix throughout the entire simulated process of digestion, followed by their slight degradation under the action of the acidic pH and pancreatic juice (Figure 4a,b, the *FCC* sample). Furthermore, for the *FCC* fraction, it can be highlighted that the carotenoids were more sensitive to the acidic pH than the alkaline one, with a degradation percentage of 13.27% in the gastric step and only 6.03% in the intestinal one. Regarding the *DCC* sample, the heat treatment conducted at a low temperature (40 °C) prevented the carotenoids from being released from the cells and, consequently, from being transformed through the in vitro gastrointestinal digestibility process. The *β*-carotene behavior within the simulated digestive process was closely similar to that observed on the total carotenoids. However, it can be considered that the *β*-carotene from the *FCC* variant was very stable during digestion (no significant differences), as only 9.46% was transformed in the gastric phase and 5.12% in the intestinal phase. 

Similar observations regarding the advantages offered by mechanical disruption or homogenization processes, releasing the carotenoids from the food matrix and making them more accessible for the digestive enzymes, were also reported previously [37,38,39]. Courraud et al. [38] studied a liquid carrot sample, carrot juice, and reported that at the end of the intestinal digestion, carotenoids presented a very low bioaccessibility, with β-carotene, as the major compound, showing a 100% recovery rate.

## 3. Materials and Methods

### 3.1. Materials

The carrots (*Daucus carota* sp.) were purchased from the local market (Galati, Romania). ABTS (2,2′-azino-bis(3-ethylbenzothiazoline-6-sulfonic acid)) and Folin–Ciocalteu reagent were purchased from Sigma-Aldrich Chemical Co. (Missouri, MO, USA), while MRS broth and MRS agar were obtained from Merck (Darmstadt, Germany).

### 3.2. Lactic Acid Bacteria Strain

*Lactobacillus plantarum* MIUG BL4 strain was used to obtain probiotic carrot-based products. The strain belongs to the Microorganisms Collection of the Bioaliment Research Platform (acronym MIUG), Faculty of Food Science and Engineering, ”Dunărea de Jos” University of Galați, Romania. The culture stock was preserved in 40% (*w*/*w*) glycerol at −80 °C for further analysis. The inoculum was obtained by cultivation on MRS (De Mann, Rogosa and Sharpe—Merck, Darmstadt, Germany) broth, for 48 h at 37 °C. The cells were centrifuged at 4800 rpm, at 4 °C, for 10 min and then washed twice with sterile 0.85% saline solution. Further, a 10^9^ CFU/mL inoculum of *L. plantarum* MIUG BL4 was used to spray the carrot-based products [25].

### 3.3. Sample Preparation

The carrots were washed and cut using a food processor under aseptic conditions into cubes (with an edge of 5 mm ± 0.2 lengths), slices, or thin chips (with an area of 2–3 cm^2^). The vegetal matrix was split into equal amounts of 100 g each, transferred to sterile Petri dishes, and exposed to UV light for 30 min, using a SafeFastElite 215S Microbiological Safety Cabinet (Faster, Cornaredo, Italy). On the fresh carrot cubes, the bacterial suspension was sprayed in a 15:1 (*w*/*v*) ratio. After that, half of these samples (*FCCs*) were packed into sterile, single-use plastic ziplock bags and refrigerated at 4 °C for microbiological analysis to determine the shelf life. The other half of the samples were frozen at −80 °C (Angelantoni Platinum 500+, ALS, Massa Martana, Italy) and then freeze-dried at 10 mBar and −50 °C for 48 h, using CHRIST ALPHA 1-4 LD plus equipment (Martin Christ, GmbH, Osterode am Harz, Germany) until a constant weight was obtained (with a 4.25 ± 0.2% water content). The freeze-dried samples were then ground, portioned, and packed identically in quantities of 10 g powder (*FDCP*) per sachet and were kept at room temperature. The chips were slowly dried (at 40 °C for 18 h) until a 12.55 ± 0.2% moisture content was reached, by using a cabinet Stericell 111 air dryer (Medcenter GmbH, München, Germany). On the dried chips, the LAB suspension (*Lactobacillus plantarum* BL4) was sprayed in a ratio of 30:1 (*w*/*v*) to obtain the dried chip (*DCCs*) variant. These samples were transferred into sterile, single-use plastic ziplock bags (IKEA, Bucharest, Romania) that were kept at room temperature to perform the microbiological analysis.

Thus, all three variants, fresh carrot cubes (*FCCs*), dried carrot chips (*DCCs*), and freeze-dried carrot powder (*FDCP*), contain LAB. In the *DCC* variant, LAB was sprayed after drying the chips; in the *FDCP* variant, LAB was sprayed before freeze-drying and grinding; and in the fresh variant (*FCCs*), LAB was sprayed on the carrot cubes and kept in this form at 4 °C.

### 3.4. The Count of L. plantarum MIUG BL4

The viability of the *L. plantarum* MIUG BL4 was evaluated weekly, by cultural methods, for 21 days. The samples were homogenized in a 0.85% sterile saline solution using Pulsifier equipment (Microgen Bioproduct, London, UK) at medium speed and maintained for 3 min. The homogenized samples were serially diluted and spread over MRS agar supplemented with 1% CaCO_3_. Then, the plates were incubated at 37 °C for 48 h and the colonies were counted. The experiment was conducted in triplicate. The viability of *L. plantarum* was expressed as the log^10^ of the mean number of colony-forming units (which were counted for each dilution in three different plates) (CFU/g).

### 3.5. Scanning Electron Microscopy (SEM)

The scanning electron microscopy technique was used in order to analyze the samples’ ultrastructure enriched with LAB. The analyzed samples were attached on aluminum stubs and gold-coated in an argon atmosphere by using an SPI Supplies (West Chester, PA, USA) sputter coater. The equipment used for the analysis was FEI Quanta 200 SEM equipment (Fei Europe B.v. Eindhoven, The Netherlands) with a plasma current intensity of 18 mA at a pressure of 6 mBar and a spot size of 10 mm as the working distance. The SEM images were taken at different magnifications between 100× and 5000×.

### 3.6. Confocal Laser Scanning Microscopy

Confocal images of the newly developed functional food products based on carrots and LAB were acquired with a Zeiss confocal laser scanning system (LSM 710), which was equipped with a diode laser (405 nm), Ar laser (458, 488, 514 nm), DPSS laser (diode-pumped solid-state—561 nm), and HeNe laser (633 nm). To observe the vegetal microstructures and the *L. plantarum* BL4 cells in detail, the Live/Dead backlight bacterial viability stain kit (Molecular Probes, Eugene, OR, USA) was used according to the manufacturer’s instructions so that only one drop was applied directly to the surface of each sample. The stain kit consisted of a mixture of two nucleic acid-binding stains: SYTO9, which stained all the viable bacteria (shown in green), while the propidium iodide stained the non-viable bacteria (shown in red), after 15 min of dark incubation [25]. The excitation and emission wavelengths were 480 and 500 nm for SYTO9 and 490 and 635 nm for propidium iodide, respectively. The samples were observed with a Zeiss Axio Observer Z1 inverted microscope equipped with a 40× apochromatic objective (numerical aperture 1.4). The 3D images were rendered and analyzed with the ZEN 2012 SP1 Black edition software. For each sample, twenty fields were evaluated, the viability counts were determined in two independent experiments, and each assay was performed in triplicate.

### 3.7. Carotenoids Quantification

To extract the pigments from the plant tissue, we used petrol ether as an extraction solvent so that the maximum content was obtained with the minimum pigment degradation. The liquid/solid ratio used in this study was 10:1 (*w*/*v*) and the mixture was further processed by ultrasonication for 30 min (MRC Scientific Instruments, Essex, UK). The extraction was performed at a constant frequency of 40 kHz and a power of 100 W. The resulting supernatant was collected and centrifuged at 6000× *g* at 4 °C for 5 min.

The extract was evaporated under a vacuum and under reduced pressure at 40 °C to dryness (AVC 2–18, Christ, UK). The carotenoid content (*CC*) of the extracts was determined spectrophotometrically [26,27] at a wavelength corresponding to the maximum absorption of each of the carotenoids with JENWAY 6505 UV/Vis equipment. 

The following formula was further used [40]:

*BC* (mg/g DW) = (A × M_W_ × V)/(ε × S_W_) × 100/DW (1)

where A is the absorption at 450 nm for total carotenoids and 470 nm for *β*-carotene, V is the total extract volume (mL), and S_W_ is the sample weight (g). To quantify the total carotenoids (*TCs*) and *β*-carotene (*BC*), the molecular weight (MW) and molar extinction coefficient (ε) of the representative compounds were undertaken, i.e., *β*-carotene (ε = 2592 L/mol × cm^−1^ in petroleum ether; MW = 536.88 g/mol). The matrices without LAB were analyzed as control samples.

### 3.8. Total Phenolic Content

The total phenolic content (*TPC*) was determined as stated by Barbu et al. [25], by using the Folin–Ciocalteu reagent and gallic acid as a standard. The carrot extracts were dissolved in 70% ethanol (1 mg/mL). Shortly after, 200 μL aliquots of the resulting solution were mixed with 125 μL of 2 N Folin–Ciocalteu phenol reagent, diluted 1:2 (*v*/*v*). After 3 min of mixing, 125 μL of 20% Na_2_CO_3_ and 550 μL of deionized water were added. The resulting mixture was kept for 30 min in the dark at room temperature; after that, the mixture was centrifuged at 8200× *g* for 10 min. The absorbance was measured at 765 nm [26,27,28]. The results were expressed as mg of gallic acid equivalents (GAE) per liter. The variants without LAB were analyzed as control samples. Each sample was measured in triplicate.

### 3.9. Proanthocyanidin Content

The proanthocyanidin content (*PC*) was determined as stated by Nguyen et al. [35], by using catechin as a standard. A 0.5 mL sample of carrot extracts was mixed with 3.0 mL of vanillin solution (4%) and 1.5 mL of concentrated HCl (37%), and then the resulting mixture was incubated at room temperature, in the dark, for 15 min. The absorbance was measured at 500 nm. The results were expressed as mg catechin equivalents (*CE*) per gram dry sample.

### 3.10. Saponin Content

The saponin content (*SC*) was determined as stated by Nguyen et al. [35], by using escin as standard. A 0.5 mL sample of carrot extracts was mixed with 0.5 mL of vanillin solution (8%) and 5.0 mL of H_2_SO_4_ solution (72%), and then the resulting mixture was incubated at 70 °C, in the dark, for 10 min. After the incubation, the samples were cooled in an ice water bath to room temperature. The absorbance was measured at 560 nm. The results were expressed as mg escin equivalents (*EE*) per gram dry sample [34].

### 3.11. Chromatographic Analysis of the Biologically Active Compounds in the Freeze-Dried Carrot Sample by UHPLC-MS/MS

The carrot extract was prepared by mixing 0.1 g of freeze-dried sample with 10 mL of methanol, followed by ultrasonication for 60 min (MRC Scientific Instruments). The resulting supernatant was collected and centrifuged at 6000× *g* at 4 °C for 5 min. The extract was filtered through 0.2 μm nylon membranes and then injected into the system. The UHPLC analysis was conducted using a Thermo Scientific Dionex Ultimate 3000 Series RS pump, a Thermo Scientific Dionex Ultimate 3000 Series TCC-3000 RS column compartment, and a Thermo Fisher Scientific Ultimate 3000 Series WPS-3000RS autosampler, all controlled by the Chromeleon Software, version 7.2 (Thermo Fisher Scientific, Waltham, MA, USA and Dionex Softron GMbH Part of Thermo Fisher Scientific, Karlsruhe, Germany). The method used in the UHPLC analysis employed a linear gradient. The mobile phase consisted of ultrapure water acidified at a ratio of 500 µL/L formic acid (at pH 2.5) and methanol as eluent B. The separation was performed on an Accucore U-HPLC Column C18 (150 × 2.1 mm, 2.6 µm) (Thermo Fisher Scientific, Germany). The gradient used in the UHPLC analysis followed several steps: 0–1 min 100% A, 1–10 min linear increase to 30% B, 10–26 min linear increase to 100% B and held for 4.0 min, 30–32.5 min decrease to 0% B at a 0.4 mL/min flow rate. For ionization, an HESI (Heated Electrospray) ion source was used with optimized parameters (sheath and auxiliary gas). The targeted compounds were separated, identified, and detected using the Q-Exactive mass spectrometer in negative mode at a 70,000 FWHM power, m/z 200, and a 100–1000 Da scan range of m/z. The Automatic Gain Control (AGC) was set to 3e6 and the injection time was 200 ms. The scan rate was set to two scans/sec, with one full scan event and five MS-MS events. The MS^2^ scan events targeted precursor ions that ranged between several 95–10,005 m/z intervals. The fragmentation took place in an HCD higher-energy collisional dissociation cell and was assessed in five separate Orbitrap scans, at a 35,000 FWHM resolving power. The collected data were evaluated using the Quan/Qual Browser Xcalibur 2.3 (Thermo Fisher, Dreieich, Germany). The mass tolerance window was set to 5 parts per million (ppm) for the two analysis modes. For the MS/MS analysis, the detection of at least two fragment ions with the appropriate ion ratio was performed by comparing to the reference standards. For the compounds for which no standard was available, the most reasonable molecular formula with the lowest mass error was sought in the chemical Chemspider database. Since many biologically active compounds have a similar chemical skeleton, the fragment ions from the MS-MS analysis were used to further confirm the chemical structure. NORMAN MassBank, mzCloudeTM Advanced Mass Spectral Database, and PubChem were used to aid in the confirmation process. ACDLabs MS Fragmenter 2019.2.1 software was employed to generate a fragmentation pattern of the identified compounds to achieve comparable results.

### 3.12. Antioxidant Activity

The antioxidant capacity was employed as described by Gheonea et al. [41], by using the ABTS^+^ (2,20 azino-bis (3-ethylbenzothiazoline-6-sulfonic acid) diammonium salt, Sigma Aldrich, Steinheim, Germany) radical method. In brief, ABTS radical cation (ABTS^+^) was produced by reacting equal volumes of 7 mM ABTS stock solution with 2.45 mM K_2_S_2_O_8_ and allowing the mixture to stand in the dark for 16 h before use. Further, aliquots of 1 mL ABTS^+^ solution were diluted with 35 mL methanol to obtain an absorbance of 1.12 ± 0.02 at 734 nm. Volumes of 2.85 mL of the ABTS^+^ solution and 0.15 mL of the extract were allowed to react for 2 h in a dark room before their absorbance at 734 nm was measured. The ABTS^+^ antioxidant activity of the samples was expressed as mM Trolox equivalents/g DW based on the calibration curve. The percent inhibition of ABTS^+^ was calculated as follows: Antioxidant activity (%) = (Absorbance of the blank − Absorbance of the sample)/Absorbance of blank) × 100. The variants without LAB were analyzed as the control samples.

### 3.13. The In Vitro Gastric and Intestinal Digestion Effect on the Total Content of Carotenoids and β-Carotene from the Carrot Products

To analyze the in vitro gastric and intestinal digestion effect of the obtained products, a static method was used as stated by Barbu et al. [25]. The products were shredded and mixed with 10 mM Tris-HCl, pH 7.7, at a ratio of 1 g of product to 10 mL of buffer solution. To thoroughly simulate the digestive conditions, a gastric mixture containing 20 mg of pork pepsin and 20 mL of HCl 1 N to reach a pH value of 2.0 was added to the initial mixture. The samples were further incubated at 37 °C on an orbital shaker (Optic Ivymen System, Grupo-Selecta, Barcelona, Spain) at 150 rpm. After the gastric step, the enzyme was inactivated, and the mixture was further subjected to the intestinal digestion step. The mixture contained 40 mg of pancreatic enzymes and 20 mL of sodium bicarbonate 1 M, and the pH was adjusted to 7.7. The determination of carotenoid content was assessed as described previously.

### 3.14. Statistical Analysis of Data

Unless otherwise stated, the data reported in this study represent the averages of a triplicate analysis (three parallel analytical trials) and were reported as mean ± standard deviation. The analysis of variance (ANOVA) (*p* < 0.05) was carried out to assess the significant differences between values and was performed with the Minitab 17 software.

## 4. Conclusions

In this research study, in all the carrot-based products, the survival of lactic acid bacteria (*L. plantarum* MIUG BL4) was below 6.0–7.0 log^10^ CFU/g after 14 days of storage, which could be appreciated as the shelf life of these functional products. The carrot samples (fresh, dried, or freeze-dried) that were enriched with *L. plantarum* MIUG BL4 displayed a high antioxidant activity. The carrot-based variants showed also increased contents of phenolic acids, flavonoids, and carotenoids for all three samples, especially for the dried carrot chips and the freeze-dried carrot powder, and a rather high content of total polyphenols, so their use as functional nutraceuticals is justified and correct. Confocal laser scanning microscopy and SEM certified the presence of the biologically active compounds that present a natural fluorescence and also the structural adherence of a lactic acid bacteria biofilm that adhered to the vegetal matrix, whereas the digestibility study suggested a controlled release of carotenoid compounds for all the samples. These types of functional carrot-based products, which contain high concentrations of bioactive compounds and lactic acid bacteria, could provide consumers with numerous health benefits through regular consumption, such as boosting the immune system and preventing several diseases. Furthermore, these products are ready for consumption, and a quantity of 100 g of these products could provide a sufficient daily intake of probiotics.

## Figures and Tables

**Table 1:** Phytochemical features of the *FCC*, *DCC*, and *FDCP* experimental variants—The experimental variants were compared to the control (fresh carrot cubes without LAB, dried carrot chips without LAB, and freeze-dried carrot powder without LAB).

Variant	*TC* (mg/g DW)	*BC* (mg/g DW)	*TPC* (mg GA/g DW)	*TFC* (mg CE/g DW)	AA (%)	PC (mg CE/g DW)	*SP* (mg EE/g DW)
Control *FCCs*	4.466 ± 0.012 ^a^	4.163 ± 0.031 ^a^	0.044 ± 0.002 ^a^	0.030 ± 0.001 ^a^	76.21 ± 0.004 ^a^	0.089 ± 0.006 ^a^	281.304 ± 0.054 ^a^
*FCC* sample	4.899 ± 0.017 ^b^	4.620 ± 0.021 ^b^	0.056 ± 0.001 ^b^	0.042 ± 0.001 ^b^	78.44 ± 0.001 ^a^	0.094 ± 0.004 ^b^	282.345 ± 0.043 ^a^
Control *DCCs*	0.222 ± 0.023 ^a^	0.210 ± 0.029 ^a^	0.082 ± 0.003 ^a^	0.028 ± 0.002 ^a^	85.23 ± 1.714 ^a^	0.138 ± 0.013 ^a^	283.13 ± 4.962 ^a^
*DCC* sample	0.534 ± 0.011 ^b^	0.492 ± 0.013 ^b^	0.097 ± 0.002 ^b^	0.041 ± 0.001 ^b^	89.02 ± 0.001 ^a^	0.33 ± 0.012 ^b^	316.459 ± 0.059 ^b^
Control *FDCP*	21.609 ± 1.673 ^a^	20.705 ± 1.501 ^a^	0.0866 ± 0.005 ^a^	0.061 ± 0.001 ^a^	81.12 ± 6.502 ^a^	0.188 ± 0.002 ^a^	372.902 ± 0.387 ^a^
*FDCP* sample	26.977 ± 0.130 ^b^	22.075 ± 0.14 ^a^	0.091 ± 0.001 ^a^	0.066 ± 0.002 ^b^	91.74 ± 0.003 ^b^	0.209 ± 0.024 ^b^	423.33 ± 0.067 ^b^

Note: *FCCs*—fresh carrot cubes; *DCCs*—dried carrot chips; *FDCP*—freeze-dried carrot powder; *TC*—total carotenoids; *BC*—*β*-carotene; *TPC*—total polyphenol content; *TFC*—total flavonoid content; *AA*—antioxidant activity; *PC*—proanthocyanidin content; *SP*—saponin content. For each experiment, the values from the same column that do not share a lowercase letter are statistically different at *p* < 0.05 based on the Tukey method and 95% confidence.

**Table 2:** Biologically active compounds (μg/g DW) analyzed by UHPLC-HESI-MS chromatographic technique.

No. Crt.	Retention Time	Compound	Chemical Structure	Molecular Mass	Concentration (μg/g DW)
1.	0.63	Inulin	C_18_H_34_O_17_	521.17235	nd
2.	1.23	Gallic acid	C_7_H_6_O_5_	169.01427	3.27 ± 0.09
3.	5.42	Catechin	C_15_H_14_O_6_	289.07176	63.45 ± 7.22
4.	6.01	Chlorogenic/neochlorogenic acid	C_16_H_18_O_9_	353.08783	148.75 ± 13.24
4.	6.24	Sinapic acid	C_11_H_12_O_5_	223.06122	nd
5.	6.41	Caffeic acid	C_9_H_8_O_4_	179.03501	16.89 ± 0.56
6.	7.86	Coumaroylquinic acid derivative	C_16_H_18_O_8_	337.09292	nd
7.	7.80	Epicatechin	C_15_H_14_O_6_	289.07176	17.3 6 ± 2.49
8.	8.71	Hidroxyferulic acid	C_16_H_20_O_10_	371.09839	nd
9.	10.01	Ferulic acid	C_10_H_10_O_4_	193.05066	85.24 ± 1.09
10.		p-coumaric acid	C_9_H_8_O_3_	163.03954	8.65 ± 0.05
11.	12.37	Rutin (quercetin 3-rutinoside)	C_27_H_30_O_16_	609.14613	102.45 ± 12.36
12.	12.78	Naringin	C_27_H_32_O_14_	579.17185	92.77 ± 5.33
13.	13.49	Luteolin-O-glucoside derivative	C_21_H_20_O_11_	447.09331	nd
14.	15.02	Quercetin	C_15_H_10_O_7_	301.03540	74.12 ± 13.99
15.	15.47	Naringenin	C_15_H_12_O_5_	271.06122	14.58 ± 3.45
16.	16.19	Procyanidine B1/B2	C_30_H_26_O_12_	577.13515	nd
17.	16.65	Kaempferol	C_15_H_10_O_6_	285.04049	54.63 ± 10.54
18.	20.56	Procyanidine	C_30_H_26_O_13_	593.13006	nd
19.	24.22	β-carotene	C_40_H_56_	537.44548	67.66 ± 8.02

nd—not determined.

## Data Availability

Data is contained within the article.
